# A preclinical model of TB meningitis to determine drug penetration and activity at the sites of disease

**DOI:** 10.1128/aac.00671-23

**Published:** 2023-11-15

**Authors:** Faye Lanni, Rosleine Antilus Sainte, Mark Hansen,, Paul Parigi, Firat Kaya, Katherine LoMauro, Bernard Siow, Robert J. Wilkinson, Sean Wasserman, Brendan K. Podell, Martin Gengenbacher, Véronique Dartois

**Affiliations:** 1 Center for Discovery and Innovation, Hackensack Meridian Health, Nutley, New Jersey, USA; 2 The Francis Crick Institute, London, United Kingdom; 3 Wellcome Centre for Infectious Diseases Research in Africa, Institute of Infectious Disease and Molecular Medicine, University of Cape Town, Cape Town, South Africa; 4 Department of Medicine, University of Cape Town, Cape Town, South Africa; 5 Department of Infectious Diseases, Imperial College London, London, United Kingdom; 6 Mycobacteria Research Laboratories, Department of Microbiology, Immunology and Pathology, Colorado State University, Fort Collins, Colorado, USA; 7 Department of Medical Sciences, Hackensack Meridian School of Medicine, Nutley, New Jersey, USA; Bill & Melinda Gates Medical Research Institute, Cambridge, Massachusetts, USA

**Keywords:** tuberculosis meningitis, rabbit model, tuberculosis immunopathology, disease progression

## Abstract

Tuberculosis meningitis (TBM) is essentially treated with the first-line regimen used against pulmonary tuberculosis, with a prolonged continuation phase. However, clinical outcomes are poor in comparison, for reasons that are only partially understood, highlighting the need for improved preclinical tools to measure drug distribution and activity at the site of disease. A predictive animal model of TBM would also be of great value to prioritize promising drug regimens to be tested in clinical trials, given the healthy state of the development pipeline for the first time in decades. Here, we report the optimization of a rabbit model of TBM disease induced via inoculation of *Mycobacterium tuberculosis* into the cisterna magna, recapitulating features typical of clinical TBM: neurological deterioration within months post-infection, acid-fast bacilli in necrotic lesions in the brain and spinal cord, and elevated lactate levels in cerebrospinal fluid (CSF). None of the infected rabbits recovered or controlled the disease. We used young adult rabbits, the size of which allows for spatial drug quantitation in critical compartments of the central nervous system that cannot be collected in clinical studies. To illustrate the translational value of the model, we report the penetration of linezolid from plasma into the CSF, meninges, anatomically distinct brain areas, cervical spine, and lumbar spine. Across animals, we measured the bacterial burden concomitant with neurological deterioration, offering a useful readout for drug efficacy studies. The model thus forms the basis for building a preclinical platform to identify improved regimens and inform clinical trial design.

## INTRODUCTION

Tuberculosis meningitis (TBM), caused by dissemination of *Mycobacterium tuberculosis* (Mtb) to the central nervous system (CNS), is the most severe and devastating form of extrapulmonary tuberculosis, with an estimated 78,200 deaths in 2019 alone (1) and an average death rate of 42% among hospitalized patients (2). Untreated, TBM is uniformly fatal. The best chance of survival following diagnosis of TBM is to initiate drug treatment prior to onset of neurological disability. However, as TBM is a paucibacillary disease that often presents with a non-specific prodrome, the diagnosis is often missed. In patients who are deemed microbiologically cured, neurological sequelae such as personality changes and cognitive impairment are common, leading to irreversibly devastating quality of life (3).

The WHO-recommended drug regimen for TBM is the same as that used for drug-susceptible pulmonary tuberculosis with a prolonged continuation phase. However, this regimen achieves suboptimal treatment outcomes for reasons that, while partially understood, require greater preclinical and clinical research. The anatomical location of the disease introduces additional barriers that drugs have to transverse in order to exert their therapeutic effects: the blood-brain barrier (BBB) and blood-cerebrospinal fluid (CSF) barrier. Additionally, persisting drug-tolerant bacterial populations may reside in these locations, as they do in pulmonary lesions (4, 5). Finally, rather than enhancing drug treatment to achieve a cure, the immune system induces an inflammatory response and initiates a cascade of pathologic mechanisms that may partially contain the disease but also cause significant brain injury (6, 7).

Several clinical trials, completed and ongoing, aim to overcome the poor performance of the first-line regimen, but unlike trials in pulmonary TB, the selection of drug regimens and doses is not informed by preclinical studies in validated animal models, highlighting the impact of infection models to prioritize regimens and dosing schedules with the best potential for cure. Adjunctive corticosteroids such as dexamethasone can reduce intra-cerebral inflammation and the associated immunopathology (8), with the potential caveat that the BBB is more permeable to rifampicin and ethambutol when inflammation is present (7, 9), and therefore, anti-inflammatory therapy may have a negative impact on drug penetration to the site of disease. To overcome suboptimal penetration of rifampicin—the pillar of first-line treatment—into CSF and brain lesions (10), high-dose intravenous and oral rifampicin has been tested in clinical trials to evaluate the tolerability and efficacy of intensified regimens (11–13), and other trials have evaluated the safety, efficacy, and drug-drug interactions of high-dose rifampicin plus linezolid with or without aspirin [(14) and NCT04145258]. New and repurposed drugs such as bedaquiline, delamanid, cycloserine, and fluoroquinolones have been evaluated for the treatment of TBM, including assessment of CSF penetration (15–19). However, the sample size was often limited, and CSF is the only CNS compartment that can be sampled in patients, whereas the pathogen is also found in brain tissue and lesions (20–23). Thus, a reproducible animal model that consistently recapitulates the major neurological and immunopathological features of human disease is needed to assess drug penetration and efficacy, prioritize promising drugs and regimens for clinical testing, guide the selection of appropriate doses, and minimize costly failures. Clinical trial data constitute a valuable translational source to validate new preclinical models.

Several animal models have been developed to test antibiotic treatments and alternative therapies for TBM (24, 25). A mouse model of TBM was developed to study drug distribution in the CNS and reports the efficacy of high-dose rifampin, supporting clinical data. Plasma and CNS ^11^C-rifampicin concentrations, measured using positron emission tomography-computed tomography (PET-CT), were consistent with rifampicin concentrations in adults and children with TBM (26). The New Zealand White (NZW) rabbit has also been used to model TBM, where CNS infection reproduces the heterogeneity of disease and neurological defects seen in humans (27). While mouse models are inherently less resource intensive and present practical advantages, adult rabbits allow for in-depth studies of disease progression and immunopathology since they afford higher resolution for spatial drug distribution studies.

In this work, we started from published rabbit models aiming to optimize disease progression and immunopathology to enable drug quantitation in CSF, brain compartments, and brain lesions that cannot be sampled in patients. To this end, we elected to work with young adult rabbits (3.5 to 4.5 months old) presenting with a brain size that allows for high-resolution drug quantitation by laser-capture microdissection. We also optimized the intra-cisternal infection method without a stereotaxic frame, adapting the method published by O’Brien et al. (28) in order to achieve close to 100% survival at the time of infection and ensure that young adult rabbits consistently developed neurobehavioral signs that recapitulate the features of human TBM. To obtain adequate disease progression within practical timeframes, we varied other parameters, including inoculum preparation and inoculum size, and developed a composite scoring system, whereby infected rabbits are engaged in drug distribution and efficacy studies once they reach a specified neurological endpoint rather than a pre-determined time point.

## RESULTS

### Defining a neurological endpoint and corresponding bacterial burden

**Fig 1:**
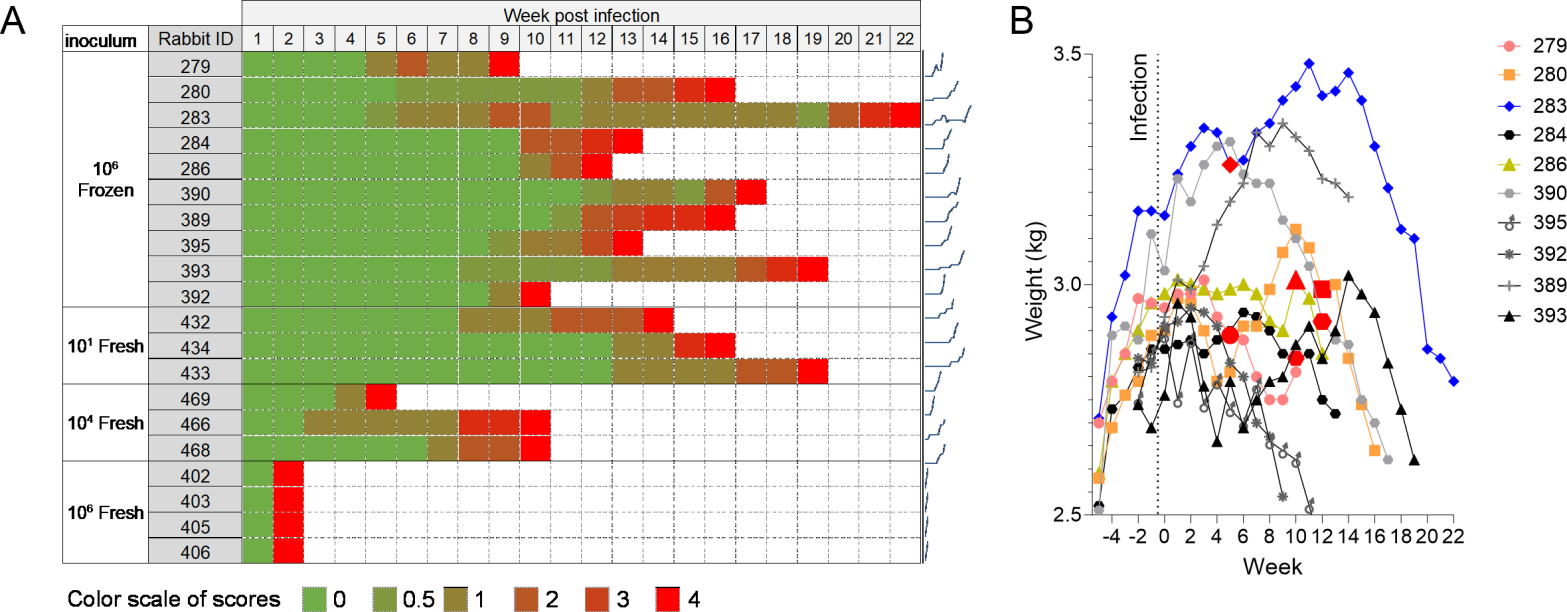
Progression of disease and neurological signs as a function of inoculum size and inoculum preparation (frozen stock or freshly grown culture). (**A**) All rabbits were infected via intra-cisternal inoculation of Mtb HN878 and necropsied when they reached a neurological score of 4. Rabbits were scored on deviations from baseline for body and head position, balance, and limb function (Table 1). Sparklines are provided to the right of each row to visualize neurological deterioration versus time. (**B**) Evolution of body weight throughout disease progression and neurological deterioration in nine rabbits infected with 10^6^ CFUs from a frozen stock of Mtb HN878 (other groups shown in Fig. S1). The time when each rabbit transited to a neurological score of 1 is indicated by enlarged red symbols.

Based on the published rabbit model of TBM using the lineage 2 Mtb strain HN878 (29), we initially infected nine rabbits intra-cisternally with 10^6^ colony-forming units (CFUs) of an exponentially grown frozen HN878 culture. Inoculation was performed via puncture of the cisterna magna circumventing the use of a stereotaxic frame (28), similar to the method used for CSF collection (30). Thus, rabbits did not undergo any procedure (i.e., attaching an acrylic helmet and tightening metallic screws) prior to infection, thereby reducing stress and opportunities for secondary infection. Also, by not positioning the rabbit in a stereotaxic frame, the infection procedure was rapidly completed, and sedated rabbits could be returned to their cages rapidly to further minimize stress. Rabbits were monitored at least weekly and were assigned a neurological score adapted from a neurobehavioral scoring scheme devised by Tucker et al. (27). We scored the body position, head position, eye opening, balance, and limb function and activity of each rabbit (Table 1). A score of 4 indicated that the rabbit had reached a humane endpoint. The highest attributed score in any category was 2 (for example, complete loss of hind limb function), and if a score of 2 was reached in any one category, this was also deemed as a humane endpoint. The most frequent signs observed in rabbits reaching a score of 4 were lack of balance, loss of limb function, eye nystagmus, and head tilt. In this initial experiment, disease progression evolved at different speeds in each of the nine rabbits, reaching a score of 4 between 9 and 22 weeks post-infection (Fig. 1A). Since weight loss is associated with active pulmonary TB disease, we recorded rabbit weights on a weekly basis. In all rabbits except #283, weight loss was concomitant with, or preceded by, neurological deterioration (Fig. 1B; Fig. S1). Weekly temperatures were recorded and remained constant throughout the course of the disease.

**TABLE 1:** Scoring system to follow disease progression and define a humane endpoint

Criteria	Score
Body position	
Elevated, normal >50% time	0
Elevated, normal <50% time	1
Continuously abnormal	2
Head position	
Elevated, normal >50% time	0
Elevated, normal <50% time	1
Continuously abnormal	2
Eye opening	
Normal	0
One eye closed, one eye open	1
Two eyes closed	2
Balance	
Normal	0
Imbalance <50% time	1
Imbalance >50% time	2
Limb functionality	
Normal	0
Monoparesis	1
Hemiparesis	2

**Fig 2:**
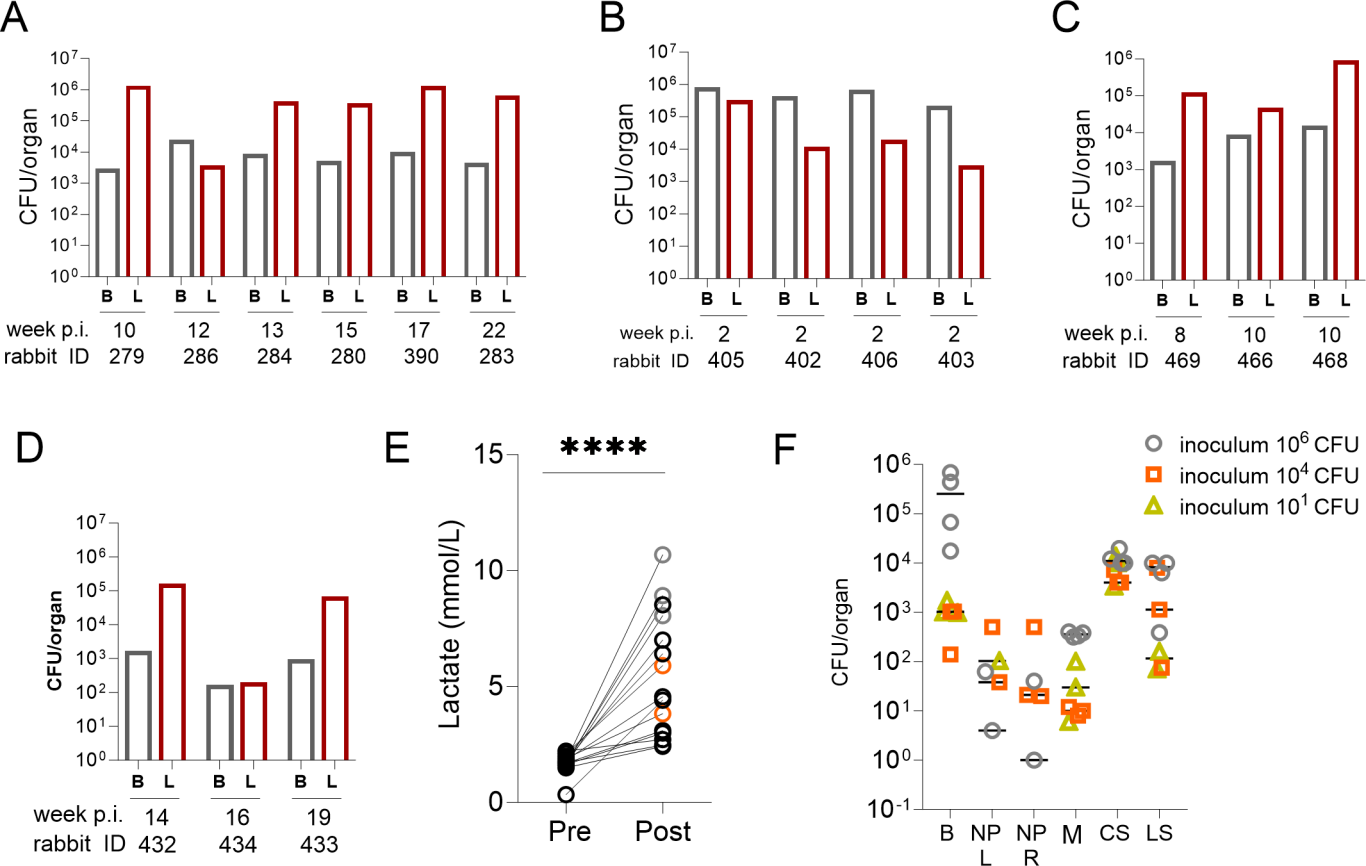
Bacterial burden in the brain and lungs of rabbits reaching a terminal neurological score. Rabbits were infected via intra-cisternal inoculation of Mtb HN878 and necropsied when they reached a neurological score of 4, independent of week post-infection. At necropsy, brains (B) and lungs (L) were removed, homogenized in PBS-T, and plated to determine colony-forming units (CFUs). Time post-infection (p.i.) in weeks and rabbit identification numbers are indicated under each pair of data points. (**A**) Brain and lung bacterial loads in rabbits that received 10^6^ CFUs of Mtb HN878 frozen stock (*n* = 6). (**B to D**) Brain and lung bacterial loads in rabbits that received 10^6^ (*n* = 4), 10^4^ (*n* = 3), and 10 CFUs (*n* = 3) of Mtb HN878 from a freshly grown culture, respectively. (**E**) CSF lactate in a rabbit subset (*n* = 15), pre-infection, and at the time a score of 4 was reached. Lactate was measured and analyzed in the EPOC handheld blood gas analyzer prior to infection (pre) and at necropsy (post). Black circles: rabbits infected with 10^6^ CFUs of frozen HN878 stock (*n* = 10); gray circles: 10^6^ CFUs of fresh HN878 culture (*n* = 3); orange circles: 10^4^ CFUs of fresh HN878 culture (*n* = 2). Due to the limited CSF volumes and the need to perform other analyses, single lactate measurements were obtained pre- and post-infection and were compared using a paired *t*-test. ****P < 0.0001. (**F**) CFU measured in distinct CNS compartments and nasal passages in a subset of rabbits. B, brain tissue (*n* = 7); NP L and R, nasal passage left and right (*n* = 6); M, meninges (*n* = 7); CS, cervical spine (*n* = 7); LS, lumbar spine (*n* = 7).

At the humane endpoint, six rabbits were euthanized to quantify bacterial burdens in CSF, lungs, and whole brains (Fig. 2A), and three rabbits were dedicated to histopathological studies, described below. No CFUs were recovered from any of the CSF samples. In this initial cohort, bacterial burdens measured in the brain and lung tissues of rabbits that had reached a score of 4 were consistent (average brain ~10^3^ CFUs and average lung ~10^5^ CFUs), independent of the time post-infection. These results suggested that the growth kinetics of the pathogen in the brain may parallel the development of neurological signs and that disease trajectories vary from animal to animal infected intra-cisternally with 10^6^ CFUs. This inter-animal variability likely reflects heterogeneous disease progression seen in patients, and faster and more synchronized appearance of neurological symptoms would be desirable to establish a practical model to study site-of-disease drug penetration and treatment efficacy.

### Optimization of disease progression

To accelerate disease progression, we hypothesized that a freshly grown culture of Mtb HN878 would exhibit higher virulence than a defrosted frozen stock (31). We infected groups of three to four rabbits with 10^6^, 10^4^, and 10^1^ CFUs from a freshly grown culture harvested in mid-logarithmic phase. The lowest inoculum of 10 CFUs was included, as it might be closest to the infectious dose that disseminates to the CNS in patients. The four rabbits that received an inoculum of 10^6^ CFUs reached a neurological score of 4 within 2 weeks (Fig. 1), with a consistently high bacterial burden in the brain (0.22 to 0.83 × 10^6^ CFUs/g tissue), higher than in the lungs (Fig. 2B). However, dissemination to pulmonary compartments was remarkably fast, reaching 10^3^ to 10^5^ CFUs within 2 weeks of intra-cisternal infection. Although disease progression was rapid and synchronized, the very short 2-week timeframe offered too limited a window for drug treatment and accurate efficacy evaluation. The three rabbits infected with 10^4^ CFUs of freshly growing Mtb reached a neurological score of 4 at week 5 (*n* = 1) and week 10 (*n* = 2) (Fig. 1). A consistently higher bacterial burden was measured in lung tissue than in brain tissue (Fig. 2C). Overall, disease progression was faster than following infection with 10^6^ CFUs from a frozen culture. However, infecting with 10^6^ CFUs from a frozen stock appears to deliver the most consistent CFUs in brains and lungs (Fig. S1), a desirable characteristic of the model for the purpose of efficacy evaluation with CFUs as one endpoint. Rabbits infected with 10 CFUs of freshly growing HN878 reached a score of 4 at weeks 14, 16, and 19 (Fig. 1), comparable to the time to reach the same score following infection with 10^6^ CFUs from a frozen stock (*P* = 0.327; Fig. S1). Likewise, pulmonary and brain burdens were comparable to the rabbit group infected with 10^6^ CFUs from a frozen stock (Fig. 2D). Thus, intra-cisternal infection with 10 CFUs from a freshly grown culture or 10^6^ CFUs from a frozen stock appears to induce similarly slow disease kinetics and neurological deterioration.

High baseline CSF lactate assists in the diagnosis of TBM and shortens the time to initiate treatment (32). We therefore investigated whether rabbits with TBM similarly present with high CSF lactate, using a handheld blood gas analyzer to quantify lactate in the CSF of rabbits pre- and post-infection. Samples were collected pre-infection when the spinal needle was inserted into the cisterna magna, and a small volume of CSF was withdrawn to check needle placement. Post-infection CSF samples were collected at the time of necropsy, following terminal sedation but prior to euthanasia, as lactate levels are known to rise post-mortem in blood. CSF lactate concentrations increased from pre-infection to the time of necropsy in all rabbits, irrespective of inoculum size and preparation (Fig. 2E).

### Quantification of bacterial burdens in central nervous system compartments

To determine how the bacterial burden is distributed within different anatomical sites of the CNS following intra-cisternal infection and to guide the selection of compartments to be sampled in drug distribution studies, we quantified CFUs in brain tissue, meninges, nasal passages, cervical spine, and lumbar spine in a subset of rabbits infected with a freshly grown Mtb culture. The spinal cord was included in these investigations since it is a common site of disease in neurological TB (33), consistent with the progressive loss of limb function observed in most rabbits. Substantial bacterial burdens (range ~10^2^ to >10^5^) were detected in the brain, cervical and lumbar spine samples, and meninges (at relatively lower numbers) (Fig. 2F). There was a trend toward higher bacterial burdens in rabbits infected with 10^6^ CFUs from a freshly grown culture, reaching a score of 4 only 2 weeks post-infection. Culturable bacteria (1 to ~500) were recovered from the majority of nasal passages. None of the CSF samples yielded any recoverable bacteria.

### Histopathology and brain lesions at the neurological endpoint

**Fig 3:**
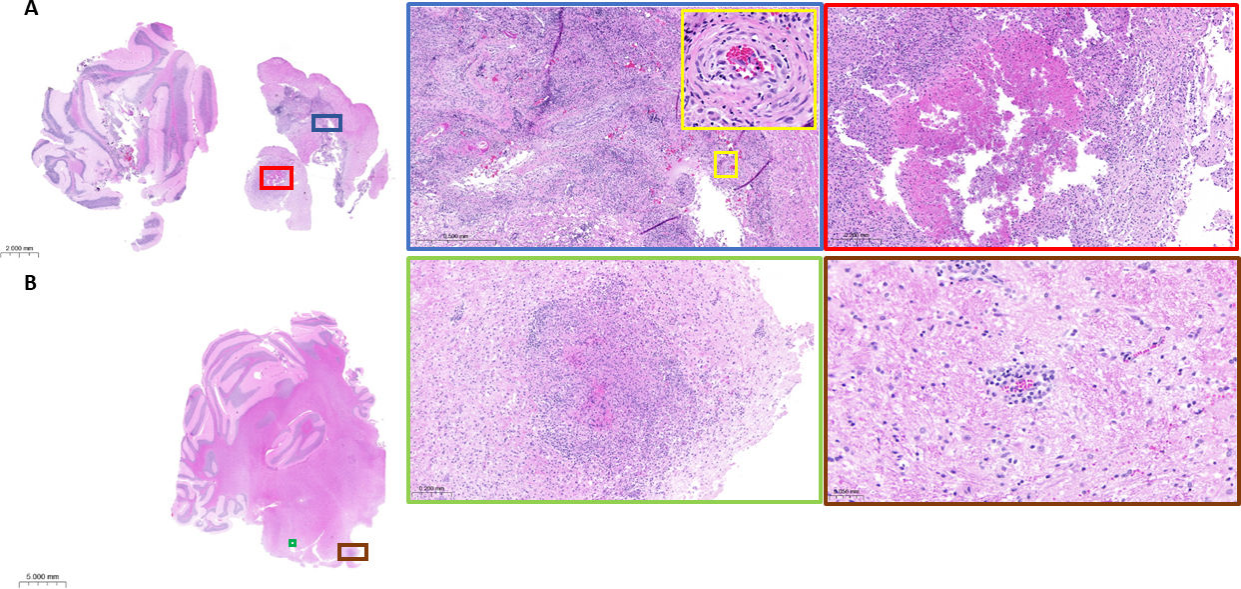
Representative H&E-stained brain sections of rabbits 280 and 466 infected with 10^6^ frozen CFUs and 10^4^ actively growing CFUs, respectively. (**A**) Rabbit 280 shows evidence of granuloma with caseation within the rostral brainstem (red square). Overlying fourth ventricle demonstrating evidence of severe lymphocytic and necrotizing vasculitis (magnified yellow square) with adventitial fibrosis and leukocyte infiltration of the tunica media, located within the choroid plexus (blue square). (**B**) Rabbit 466 shows a granulomatous lesion with caseation, similar to rabbit 280. Surrounding areas of granulomas, vessels within white matter often show evidence of perivasculitis.

**Fig 4:**
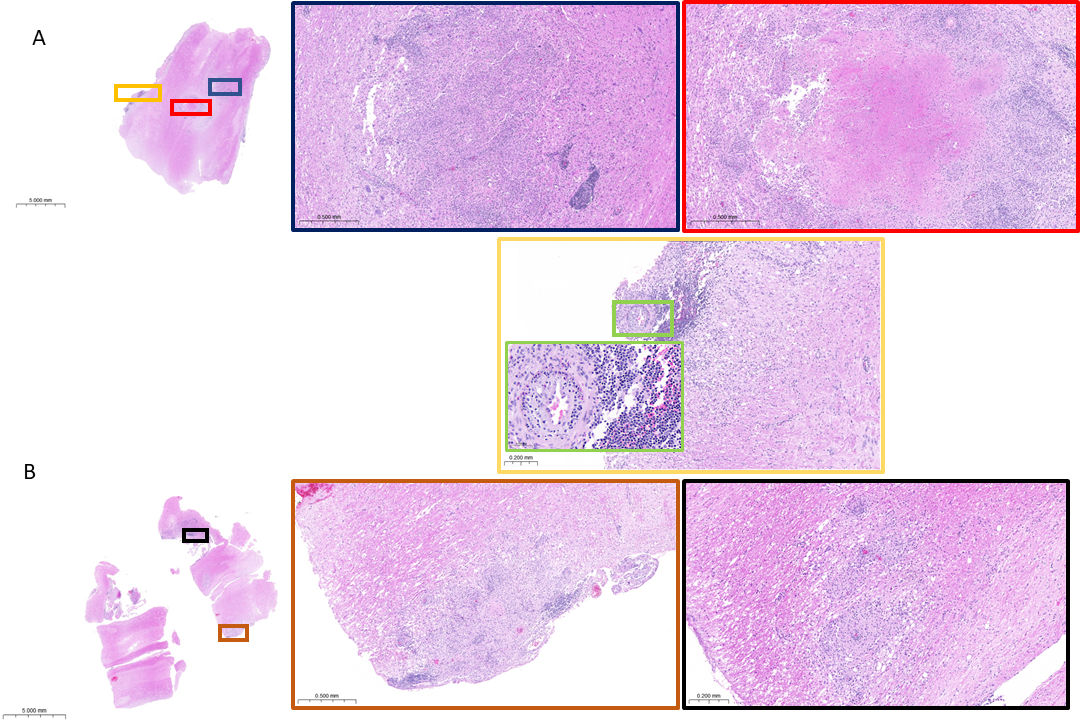
Representative H&E-stained cervical spinal cord sections. (**A**) Rabbit 393 granulomas, both cellular and caseating, are located within the spinal cord parenchyma and located at the meningeal surface. Lymphocytic infiltration surrounds areas of leukocytoclastic vasculitis (magnified green square). (**B**) Rabbit 466 shows cellular granulomas within the parenchyma and at the meningeal surface.

To relate the neurological and microbiological observations to CNS immunohistopathology, tissue sections were obtained from a subset of rabbits and processed for histological [hematoxylin and eosin (H&E)] and acid-fast [Ziehl-Neelsen (ZN)] staining. Rabbits from the “10^6^ CFU frozen” (most consistent CFU burdens) and “10^4^ CFU fresh” (faster disease progression) groups were selected to determine whether major histopathological differences were present between these groups. Representative brain slices (slice 5, encompassing the cortex, subcortical white matter, hypothalamus, thalamus, and hippocampus) (Fig. S2) from rabbits 280 and 466 infected with frozen (10^6^ CFUs) and fresh (10^4^ CFUs) cultures were collected for histopathological analysis when these two rabbits reached a neurological score of 4 at 15 and 10 weeks post-infection, respectively. Granulomatous lesions, in rabbits 280 and 466, were identified both within the rostral brainstem and cerebellum. These lesions either consist of cellular admixture of lymphocytes and macrophages or have organized lymphocytic inflammation surrounding a central area of caseous necrosis. Consistent with the route of intra-cisternal inoculation, lesions were identified within the fourth ventricle and associated choroid plexus. These cellular lesions, composed of lymphocytic inflammation and macrophages, surround areas of vasculitis (Fig. 3). Similar to those found in the cerebellar region, both cellular and caseating granulomas were present within the spinal cord parenchyma and at the meningeal surface, in both rabbit 393 infected with frozen (10^6^ CFUs) and rabbit 466 infected with fresh (10^4^ CFUs) cultures. Confirmation of lesion-associated bacilli was achieved, in both the brain and cervical spinal cord, by acid-fast staining, where the majority of bacilli were located within caseous necrosis (Fig. 4). Cellular and caseous lesions were found in the lungs of all rabbits, regardless of inoculum size and preparation (Fig. S3).

### The rabbit model of TBM to study drug penetration in the CNS

**Fig 5:**
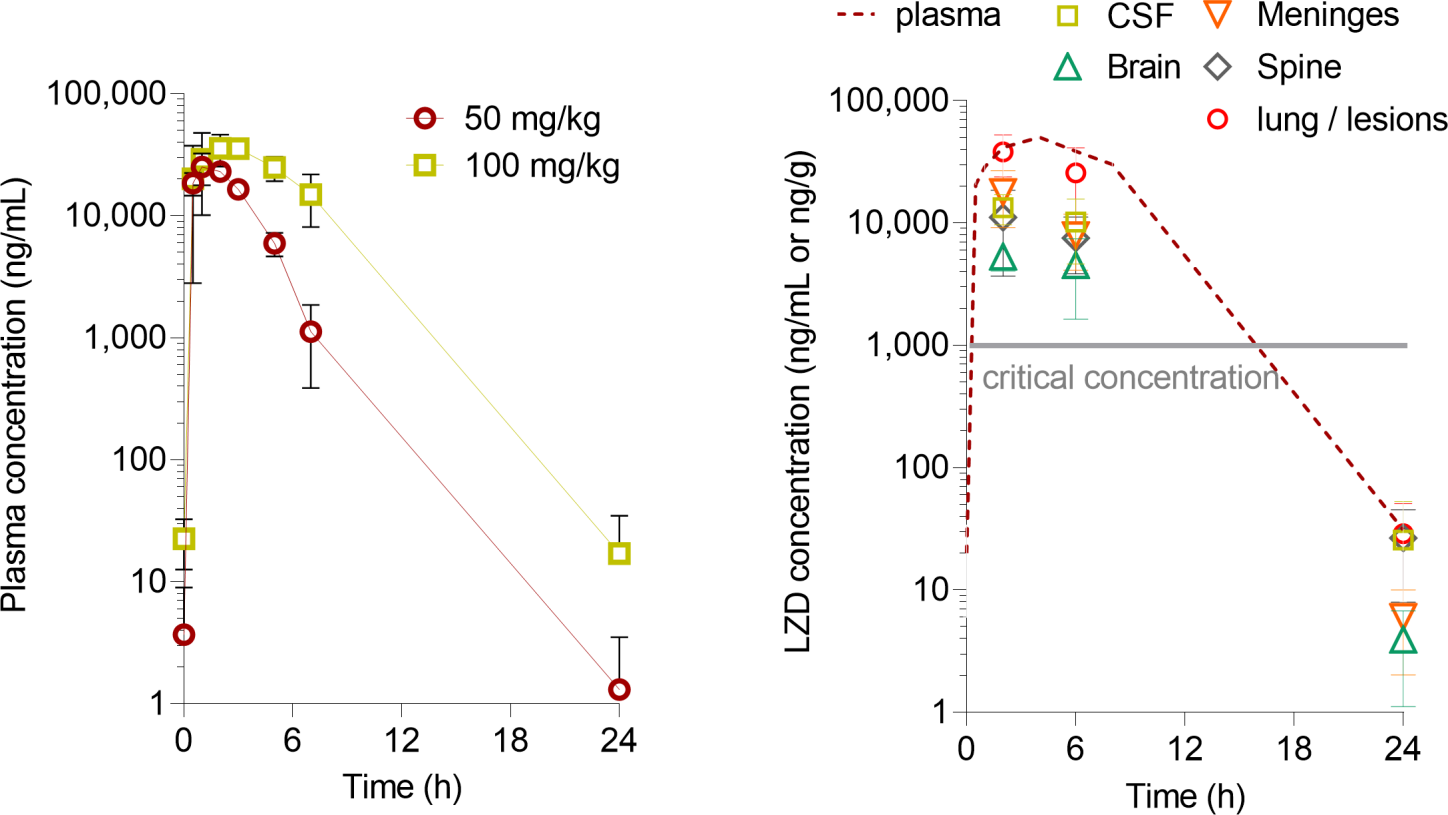
Penetration of linezolid from plasma to CNS compartments at the human equivalent dose. (**A**) Dose-finding pharmacokinetic studies in uninfected rabbits (50 mg/kg: *n* = 6; 100 mg/kg: *n* = 3). (**B**) Linezolid concentrations in the plasma, CNS compartments, and lung or lung lesions during the absorption phase (2 h post-dose) and the distribution phase (6 h) and at trough (24 h) following three daily doses of 90 mg/kg, corresponding to 1,200 mg daily in TB patients. *n* = 3 to 8 samples per data point.

Having established a scoring system that allowed for reproducible organ sampling consistent with the disease state and bacterial load, we designed macrodissection and laser-capture microdissection (LCM) schemes for spatially resolved drug quantitation in relevant CNS compartments. We first performed a linezolid dose-finding study in uninfected rabbits (Fig. 5A) and identified 90 mg/kg as the dose that reproduces clinical exposure at 1,200 mg daily, the dose in use in ongoing TBM clinical trials during the intensive phase (14, 34). Next, 12 rabbits were infected with 10^4^ CFUs from a fresh culture. When they reached a score of 3, they received three daily linezolid doses of 90 mg/kg to achieve a steady state. Groups of rabbits were analyzed after the third dose as follows: four rabbits at 2 h during the absorption phase, five rabbits at 6 h toward the end of the distribution phase, and three rabbits at 24 h or at trough. Linezolid concentrations were measured in plasma pre-dose and at 0.5, 1, 2, 4, 6, 8, and 24 h on day 1 and after the last dose or until the time of necropsy, at which point the CSF, brain, meninges, spine, lung, and lung lesions were collected, weighed, homogenized (except for CSF), and stored at −80°C until analyzed. A subset of brain samples was sliced (Fig. S2) prior to homogenization or LCM for spatial quantitation of linezolid. We found that concentrations of linezolid were three- to fourfold lower in the CSF, meninges, and spine than in matched plasma and lower in brain tissue than in other CNS compartments (Fig. 5B). In one rabbit analyzed at 6 h after the last dose, concentrations in all brain slices were similar: range 3,950 to 4,320 ng/g (SD 147 ng/g). In each compartment, observed concentrations were above the critical concentration of 1.0 µg/mL for pulmonary TB (35) for at least half of the dosing interval. Linezolid CSF/plasma ratios measured in this study (0.32, 0.26, and 0.80 at 2, 6, and 24 h, respectively) were similar to adjusted CSF/serum ratios measured in TBM patients (0.25 and 0.59 at 2 and 6 h, respectively) (15).

## DISCUSSION

We optimized an animal model of TBM in young adult rabbits, reproducing several features typical of clinical TBM: neurological deterioration, disease progression leading to a terminal neuropathological score but with marked interindividual variability in the dynamics of disease development, acid-fast bacilli seen in the context of necrotic lesions in the brain or spinal cord (36), and elevated CSF lactate (37). None of the infected rabbits recovered or controlled the disease. The signs of neurological deterioration included limb hemiparesis or paraparesis, neck stiffness, and eye movement disorders, as seen in TBM patients. The timing of disease progression and neurological deterioration over 5 to 20 weeks post-infection was also consistent with human TBM, which typically presents as subacute illness (7).

To ensure reproducibility of sampling and analyses across studies, we developed a composite scoring system that integrates neurological symptoms typical of TBM. CNS bacterial burdens measured at the humane endpoint, i.e., when the animals reached a maximum neuropathological score of 4, were consistent across rabbits. The onset of neurological deterioration also correlated with weight loss. These features enable evaluation of treatment efficacy and studies of drug penetration at consistent time points in relation to disease stages across animals. Thus, outbred New Zealand White rabbits present with expected inter-animal variability in disease progression but not to the extent that this variability prevents practical use of the model as an experimental tool. Rabbits are amenable to high-resolution omics studies given their large size, offering the opportunity to compare proteome-, genome-, and metabolome-wide profiling with clinical and mouse data (38–40).

Disease progression was markedly accelerated when rabbits were infected with a freshly grown culture rather than a frozen stock (2 weeks versus 9 to 22 weeks with an inoculum of 10^6^ bacteria). This has been previously observed in the rabbit model of active pulmonary TB, where a large, inhaled dose of frozen-thawed bacilli produced fewer tubercles than a much smaller inhaled dose of log-phase bacilli. The phenomenon has been attributed to a lag phase of frozen-thawed bacilli, during which they may be less able to survive phagocytes than actively growing log-phase bacilli (31). Actively growing Mtb may also be able to resume multiplication sooner after infection, leading to faster dissemination from the cisterna magna injection site through the brain. Comparing the CFU burden in the brain and lungs of the four inoculation groups revealed that infecting with 10^6^ CFUs from a frozen stock appears to deliver consistent CFUs in both organs (Fig. S1), amenable to efficacy studies with CFUs as one of the readouts. Since starting from frozen stocks also facilitates reproducibility across experiments, this size and type of inoculum will preferentially be adopted in future efficacy experiments. We acknowledge that the rabbit model presents limitations as a tool for efficacy studies: (i) amounts of drugs needed given the large animal size, (ii) large number of animals required when comparing multi-drug treatments, and (iii) limited plasma PK data compared to available literature in mice, although the pharmacokinetic profiles and human-equivalent doses of most TB drugs have been published by our group in the context of pulmonary distribution studies or can be shared upon request if unpublished. For pharmacokinetic studies focusing on drug concentrations in lesions and brain compartments, infecting with 10^4^ CFUs from a fresh culture is adequate and potentially more economical since (i) disease progression appears faster and well synchronized and (ii) histopathological features are similar in rabbits infected with 10^6^ CFUs from a frozen stock or 10^4^ CFUs from a fresh culture (Fig. 3 and 4).

The CNS bacterial load was distributed across several compartments surveyed, including all brain slices from the frontal cortex to the cerebellum, meninges, cervical spine, and lumbar spine. Distribution of CSF from the site of cisterna magna infection along the spinal cord is well described and has been exploited for gene delivery to the brain and spine (41). In addition, smaller numbers of bacilli were recovered from the nasal passages. Although the mechanism was not determined, this is potentially associated with the CSF outflow pathway along the olfactory sensory nerves through the cribriform plate and into the nasal epithelia (42, 43) or the nasal lymphatics (44, 45). The bacterial burden was highest in the lungs, except in rabbits infected with a high dose of freshly grown Mtb cultures, whose disease culminated within 2 weeks, at which point the disease burden was not surprisingly still higher in the CNS than in pulmonary compartments. Thus, in our model of cisterna magna infection, the pathogen spreads from the CNS to the lungs, in contrast to the presumed pathway in TBM patients, where bacteria are thought to disseminate to the brain and spine from another site, originating in the lungs. This observation suggests that Mtb can travel in forward and reverse directions across the BBB in the rabbit model, a hypothesis that could be considered in TB patients. Extra-CNS dissemination to the lungs and other organs after direct brain infection in animal models has been described previously (46, 47), attributed to the breakdown of the BBB due to inflammation.

In zebrafish and *ex vivo* studies, *Mycobacterium marinum* was found to cross the BBB via two different mechanisms, a Trojan horse mechanism exploiting phagocytes, as well as invasion and infection of endothelial cells, with subsequent damage of the basal lamina (48). All rabbits surveyed in this work acquired pulmonary TB following CNS infection, which cannot be attributed to rabbit-to-rabbit transmission since single animals are housed in individually ventilated cages. In contrast, lung-to-brain dissemination has not been observed in rabbits and is mostly—but not exclusively—limited to children and HIV-positive patients. CSF drains through arachnoid granulations into the superior sagittal sinus and is returned to venous circulation (49, 50). Indeed, bacterial infection has been shown to spread from the meninges to the superior sagittal sinus via the diploic veins, which ultimately drain into the jugular vein. Whether the dominant route of dissemination observed in this model is hematogenic, lymphatic, or via the airways and whether the nasal passages play a role in dissemination remain to be determined and could contribute to a better understanding of the lung-brain axis of TB dissemination.

We did not recover culturable bacteria on solid Middlebrook 7H11 medium in any of the CSF samples. While positive CSF culture or microscopy constitutes a definite TBM diagnostic (36), negative CSF culture is frequent in TBM patients with ~30% sensitivity for conventional culture (51) and lower in children. In a pediatric study seeking to establish a diagnostic rule (52), only 18 out of 110 patients were CSF positive for Mtb (by mycobacterial culture or acid-fast bacilli staining), whereas the remaining patients were diagnosed on clinical and radiological grounds. Thus, our findings mirror the clinical experience and may support a route of dissemination that does not involve the CSF, either hematogenic or via the nasal passages, consistent with the lower numbers of bacilli in the meninges than in deeper brain structures. Alternative growth medium and molecular methods will be included in future studies to increase the sensitivity and likelihood of detecting viable Mtb in rabbit CSF.

We found lesions within parenchymal tissue and at the meningeal surface of both the brain and cervical spinal cord, highlighting the importance of quantifying drug concentrations in all regions of the CNS. To evaluate the suitability of magnetic resonance imaging (MRI) as a modality to visualize and locate abnormalities within whole brains inside their skull environment, rabbit 389 (infected with 10^6^ frozen CFUs and reaching a neurological score of 4 at 16 weeks post-infection, Fig. 1) was dedicated to MRI scanning *ex vivo*, without the use of a contrast agent. Abnormalities were detected in the hind brainstem and the cervical spine, including tuberculoma-like lesions typical of clinical TBM (53–55) (Fig. 3). These *ex vivo* images support MRI of live animals with the use of a contrast agent as a promising 3D imaging platform to localize lesions within the CNS, characterize human-like features of the model, follow disease progression, and monitor treatment response. MRI plays a major role in the detection of brain abnormalities related to TBM and offers a non-invasive soft tissue contrast imaging modality with high spatial resolution without ionizing radiation (56).

The finding that linezolid levels are lower in the CNS than in plasma, as well as the differences between CSF and brain linezolid levels, is consistent with published studies using direct drug measurements and PET-CT imaging (57, 58). Linezolid CSF/plasma ratios were similar to adjusted CSF/serum ratios measured in TBM patients (15), supporting the model’s translational value for drug penetration studies in the CNS. Similar studies with first- and second-line TB drugs are in progress to validate the model. As our understanding of the optimal site of drug action in TBM improves, the rabbit model provides an ideal tool to quantify drug distribution with adequate spatial resolution in all clinically relevant areas from white matter found in deep regions of the cerebrum and thalamus, granular and molecular layers of the cerebellum, to regions classically associated with TBM disease: CSF, meninges, base of the brain around the circle of Willis, and brainstem (20, 23).

In summary, we have developed an adult rabbit model of TBM that recapitulates the major known sites of clinical TBM disease. We have exploited the model to study the penetration of linezolid into the CNS. Combining drug quantitation by conventional high pressure liquid chromatography (HPLC) coupled to tandem mass spectrometry in fluids and tissue homogenates with LCM in thin brain sections, we can deliver absolute drug concentrations with rich spatial information in any part of the CNS considered to be clinically relevant, whether for antibacterial or host-directed therapeutic intervention.

## MATERIALS AND METHODS

### Preparation of HN878 for rabbit infection

*M. tuberculosis* strain HN878 was cultured in Middlebrook 7H9 broth supplemented with 10% oleic acid-albumin-dextrose-catalase (Becton Dickinson), 0.2% glycerol, and 0.05% Tween_80_. Cultures were grown to mid-log phase (OD_600nm_ 0.4–0.7). On the infection day, OD_600nm_ was measured after passing the culture through a TB insulin syringe (27G) 10 times to remove bacterial aggregates. The culture was centrifuged, and bacteria were resuspended in phosphate-buffered saline (PBS) to a final concentration of 2 × 10^6^ CFUs in a volume of 100 µL for the first round of infection (10^6^ CFUs per rabbit in a volume of 50 µL) or diluted as appropriate to achieve the target number of CFUs/rabbit in 50 µL. The OD_600nm_ was confirmed and CFU enumerated. For subsequent rounds of infection with frozen stocks, aliquots of mid-log phase HN878 stored at −80°C were defrosted and diluted as appropriate in PBS to infect rabbits with various inoculum sizes as described. For the infection group that received an inoculum target of 10 CFUs per rabbit, the actual infection dose, as measured by plating on solid medium, was 14 ± 3 CFUs.

### Infection method

An adaptation of the blind puncture of the cisterna magna without stereotaxic equipment was used to infect rabbits in these studies (29). Female New Zealand White rabbits (Charles River Laboratories) weighing 2.2 to 2.6 kg were maintained under specific pathogen-free conditions and fed water and chow *ad libitum*. Rabbits were anesthetized with a combination of 2 mg/kg xylazine and 25 mg/kg ketamine. Rabbits were placed in lateral recumbency with the head flexed at an angle of 90°. The external occipital protuberance was used as a landmark, and a human lumbar puncture needle (Spinal Needle BD Quincke Style 25 Gauge 1 Inch Short, Neonatal Type) was inserted into the cisterna magna. Once placement of the needle in the cisterna magna was confirmed, a baseline CSF sample (approximately 100 µL) was drawn into an empty syringe. Next, the syringe was swapped with the syringe containing the Mtb inoculum. Precaution was taken to ensure that neither the animal nor the needle was allowed to move during the syringe swap to avoid needle-induced injuries. A volume of 50 µL of Mtb HN878 inoculum was injected over a period of 20 seconds.

### Measurement of CSF lactate

At the time of infection, a 100-µL sample of CSF withdrawn to ensure correct needle placement was recovered, stored, and analyzed on an EPOC handheld blood gas analyzer (Element POC, HESKA, CO, USA).

### Neurological scoring

Rabbits were scored weekly for neurological signs based on the scoring system described (27), with the simplification that body position, head position, balance, and limb functionality and activity were the only categories scored (Table 1). The highest obtainable score in any given category was 2, and rabbits were euthanized if a score of 2 was recorded in a single category or if the combined score of all categories reached or exceeded 4.

### Drug administration for tissue distribution studies

At a neurological score of 3, rabbits received three daily doses of 90 mg/kg linezolid formulated in 0.5% carboxymethyl cellulose (CMC)/0.5% Tween 80 administered by oral gavage. Blood was collected from the central ear artery pre-dose and at 0.5, 1, 2, 3, 5, 7, and 24 h post-drug administration on days 1 and 3. Rabbits were necropsied, with a terminal CSF sample collected at either 2, 6, or 24 h after the third and last dose.

### Necropsy

Rabbits were euthanized (for bacterial burden, histology, and other disease-related studies) when a neurological score of 4 was reached or following 3 days of drug administration (for drug penetration studies). Prior to necropsy, rabbits were terminally sedated with a combination of 5 mg/kg xylazine and 35 mg/kg ketamine, and terminal CSF and cardiac blood were collected. Following this, a lethal dose of sodium pentobarbitone (Euthasol) was administered, and the brain, meninges, nasal swabs, cervical and lumbar spinal cord, lung, liver, and spleen were collected for bacterial load enumeration and histopathological evaluation. Brains were sliced using an egg slicer (OXO Good Grips Egg Slicer, Amazon), and the same slice was taken from each rabbit for H&E and ZN staining. The remaining brain was homogenized in 12.5 mL of phosphate-buffered saline supplemented with 0.5% Tween 80 (PBS-T); the spinal cord and meninges were homogenized in 1 mL of PBS-T, and all other organs were homogenized as previously described using an IKA homogenizer (Ultra Turrax Tube Drive, Fisher Scientific). Homogenized organs were serially diluted or plated in entirety on Middlebrook 7H11 agar supplemented with bovine albumin and catalase. Plates were incubated for 4 to 5 weeks; colonies were enumerated, and results are expressed as the number of CFUs per organ.

Sections of the brain, meninges, cervical spinal cord, lung, liver, and spleen were also collected for drug quantitation via high-pressure liquid chromatography coupled to tandem mass spectrometry (LC/MS-MS) and processed as described below.

### Linezolid quantitation in plasma and tissue homogenates

Linezolid was purchased from Sigma-Aldrich. The linezolid-d8 internal standard was purchased from Toronto Research Chemicals. The drug-free K_2_EDTA plasma, CSF, liver, spleen, lungs, and brain from NZW rabbits were obtained in-house and from BioIVT for use as blank matrices to build standard curves. A neat 1 mg/mL linezolid DMSO stock was serially diluted in 50/50 acetonitrile (ACN)/water to create standard curves and quality control spiking solutions. Spiked matrix standards and quality control (QC) samples were created by adding 10 µL of spiking solutions to 90 µL of drug-free plasma, CSF, or control tissue homogenates. Extraction was performed for standards, QC, and study samples by adding 200 µL of 1:1 acetonitrile (ACN)/methanol (MeOH) containing 40 ng/mL stably labeled linezolid-d8 to 20 µL of plasma, CSF, or homogenized tissue samples and 20 µL of 1:1 ACN:H_2_O.

LC/MS-MS analysis was performed on a Sciex Qtrap 6500+ triple-quadrupole mass spectrometer coupled to a Shimadzu Nexera X2 UHPLC system to quantify linezolid in plasma. Chromatography was performed on an Agilent Zorbax SB-C8 column (2.1 × 30 mm; particle size, 3.5 µm) using a reversed-phase gradient elution with aqueous. Milli-Q deionized water with 0.1% formic acid (FA) was used for the aqueous mobile phase and 0.1% FA in ACN for the organic mobile phase. Multiple reaction monitoring (MRM) of precursor/fragment transitions in electrospray positive-ionization mode was used to quantify the analytes. MRM transitions of 338.00/235.00 and 346.15/304.20 were used for linezolid and linezolid-d8, respectively. Sample analysis was accepted if the concentrations of the quality control samples were within 20% of the nominal concentration. Data processing was performed using Analyst software (version 1.6.3; Sciex).

### Laser-capture microdissection

Twenty-five-micrometer-thick tissue sections were cut from rabbit lung and brain biopsies using a Leica CM 1860UV (Buffalo Grove, IL, USA) and thaw-mounted onto 1.4-µm-thick Leica PET-Membrane FrameSlides (Buffalo Grove, IL, USA) for laser-capture microdissection. Tissue sections were immediately stored in sealed containers at −80°C. Adjacent 10-µm-thick tissue sections were thaw-mounted onto standard glass microscopy slides for H&E and ZN staining. Cellular, necrotic (caseum), uninvolved lung lesion areas for the lung and mesencephalon and cortex areas for the brain totaling 3 million µm^2^ were dissected from three to five serial biopsy sections. The total tissue volume of each pooled sample was determined based on the surface area of the pooled sections and the 25-µm tissue thickness. Areas of interest were identified optically from the brightfield image scan and by comparison to the adjacent H&E reference tissue. Pooled dissected tissues were collected into 0.25-mL standard PCR tubes and immediately transferred to −80°C.

Two microliters of neat spiking solutions was added to 2 µL of tissue homogenates prior to extraction. Two microliters of ACN:H_2_O and 2 µL of PBS were added to the dissected study samples. Extraction was performed by adding 50 µL of extraction solution ACN/MeOH (1/1) with 40 ng/mL linezolid-d8. Extracts were vortexed for 5 minutes and centrifuged at 10,000 rpm for 5 minutes. Forty microliters of supernatant was transferred for LC/MS-MS analysis, diluted with an additional 40 µL of Milli-Q water, and processed as described above for linezolid quantitation.

## SUPPLEMENTAL MATERIAL

MRI methods, Supplemental figures 1 to 4 aac.00671-23-s0001.pdf MRI methods. Fig. S1 related to Fig. 1. Fig. S2: CNS tissue processing post-necropsy for evaluation of bacterial burden, histopathology, and spatial drug quantitation. Fig. S3 showing pulmonary histology findings. Fig. S4 showing *ex vivo*, non-contrast-enhanced MRI of the cranial area, brainstem, and cervical spine.

ASM does not own the copyrights to Supplemental Material that may be linked to, or accessed through, an article. The authors have granted ASM a non-exclusive, world-wide license to publish the Supplemental Material files. Please contact the corresponding author directly for reuse.
