# Supplementary material for: A preclinical model of TB meningitis to determine drug penetration and activity at the sites of disease
Source: Antimicrob Agents Chemother. 2023 Nov 15;67(12):e00671-23. doi: 10.1128/aac.00671-23 (PMC10720511; doi:10.1128/aac.00671-23)
Supplement: MRI methods, Supplemental figures 1 to 4 — MRI methods. Fig. S1 related to Fig. 1. Fig. S2: CNS tissue processing post-necropsy for evaluation of bacterial burden, histopathology, and spatial drug quantitation. Fig. S3 showing pulmonary histology findings. Fig. S4 showing ex vivo, non-contrast-enhanced MRI of the cranial area, brainstem, and cervical spine. [file aac.00671-23-s0001.pdf]

A preclinical model of TB meningitis to determine drug penetration and activity at the sites of disease.

Faye Lanni [1] ¥, Rosleine Antilus Sainte [1], Mark Hansen Jr [1], Paul Parigi [1], Firat Kaya [1], Katherine LoMauro [1], Bernard Siow [2], Robert J. Wilkinson [2,3,4], Sean Wasserman [3], Brendan Podell [5], Martin Gengenbacher [1,6] and Véronique Dartois [1,6] *.

Running title: A preclinical model of TB meningitis.

*Corresponding author

Affiliations

[1] Center for Discovery and Innovation, Hackensack Meridien Health, 111 Ideation Way, Nutley, NJ

[2] The Francis Crick Institute, 1 Midland Road, London, UK. NW1 1AT

[3] Wellcome Centre for Infectious Diseases Research in Africa, Institute of Infectious Disease and Molecular Medicine and Department of Medicine, University of Cape Town, Observatory 7925, Republic of South Africa

[4] Department of Infectious Diseases, Imperial College London W12 0NN, United Kingdom

[5] Mycobacteria Research Laboratories, Department of Microbiology, Immunology and Pathology, Colorado State University, Fort Collins, Colorado, USA

[6] Department of Medical Sciences, Hackensack Meridien School of Medicine, 123 Metro Boulevard, Nutley, NJ

¥ Present address: Mycobacteria Research Laboratories, Department of Microbiology, Immunology and Pathology, Colorado State University, Fort Collins, Colorado, USA

Keywords: tuberculosis meningitis, rabbit model, tuberculosis immunopathology, disease progression

Supplemental Methods

Magnetic resonance imaging (MRI)

Sample preparation: Whole rabbit heads were collected and all fur and tissue, including the ears and lower jaw, were removed, but the eyes remained in situ. The skulls were placed in 500 mL of formaldehyde and shipped to the UK for MRI scanning. Immediately prior to scanning, samples were wrapped in a single layer of gauze and were placed on Press'n Seal® Cling Film (The Glad Products Company; Oakland CA, U.S.A.). A custom pocket was formed around the head by pressing firmly on the film and the pocket was filled with 10% neutral buffered formalin (CellSolv, CellPath Ltd; Powys, UK). The pocket was then sealed around the head taking care to exclude large air bubbles. The sealed pocket was placed in a wide neck screw top plastic jar for scanning. Ex-vivo MRI scans were performed on a 9.4T 20 cm bore MRI scanner (Bruker Biospec; Ettlingen Germany) equipped with an 86 mm quadrature r.f. volume coil. Paravision 7.0.0 software (Bruker; Ettlingen Germany) was used for scan planning and acquisition. 3D Multiple gradient echo (MGE) scans were performed with the following parameters: for 250 μm scans: TR = 50 ms, 8 echoes, first echo time = 3 ms, echo spacing = 3.644 ms, FA = 12.8°, 80x64x32 mm^3 FoV, 320x256x128 matrix, 12 averages, scan duration: 5h27m; for 200 μm scans: TR = 50 ms, 8 echoes, first echo time = 3 ms, echo spacing = 3.7 ms, FA=12.8°, 64x64x32 mm^3 FoV, 320x320x160 matrix, 12 averages, scan duration 8h32m; for 100 μm scans: TR = 75 ms, 4 echoes, first echo time = 4.5 ms, echo spacing = 7 ms, FA=12.8°, 64x64x24 mm^3 FoV, 640x640x240 matrix, 11 averages, scan duration 35h12m.

Supplemental Figures

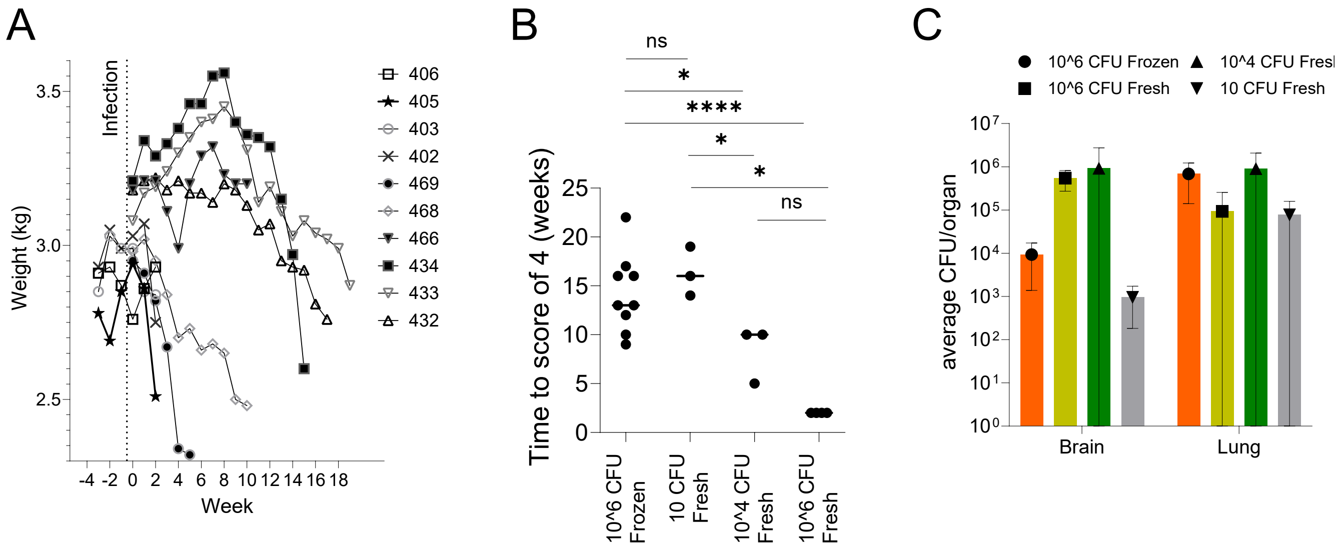

Supplemental Figure 1. Impact of inoculum size and culture conditions on the kinetics of weight loss (A), time to reach a neurological score of 4 (B) and bacterial burden in brain and lungs (C). Data in (B) were analyzed using unpaired t-test with Welch correction and individual variance for each group. Ns: non significant, * $p < 0.05$, **** $p < 0.00005$. Sample sizes in (C) are as follows: 10^6 CFU frozen: n=6; 10^6 CFU fresh: n=4; 10^4 CFU fresh: n=4; 10 CFU fresh: n=3.

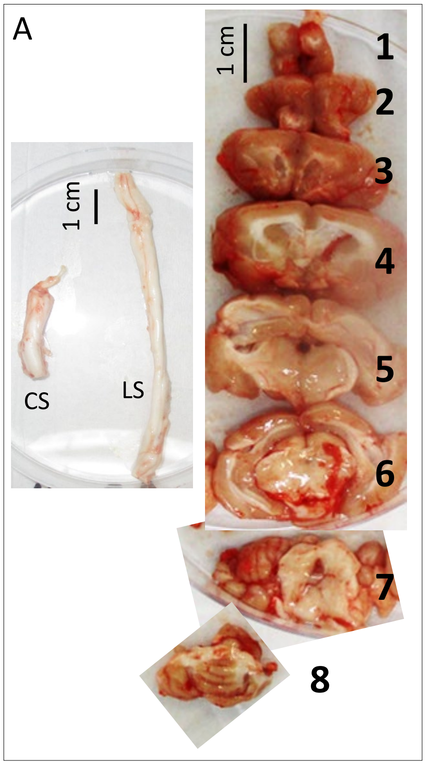

B

Brain slice	Key features
1	Olfactory bulbs, frontal cerebral cortex
2	Frontal cortex
3	Cingulate cortex, internal capsule
4	Corpus callosum, centrum semivale, putamen, lateral ventricles
5	Parietal cortex, subcortical white matter, hippocampus, hypothalamus
6	Occipital cortex, rostral culliculus
7	Cerebellum, ansiform lobe, paraflocculus lobe
8	Cerebellum

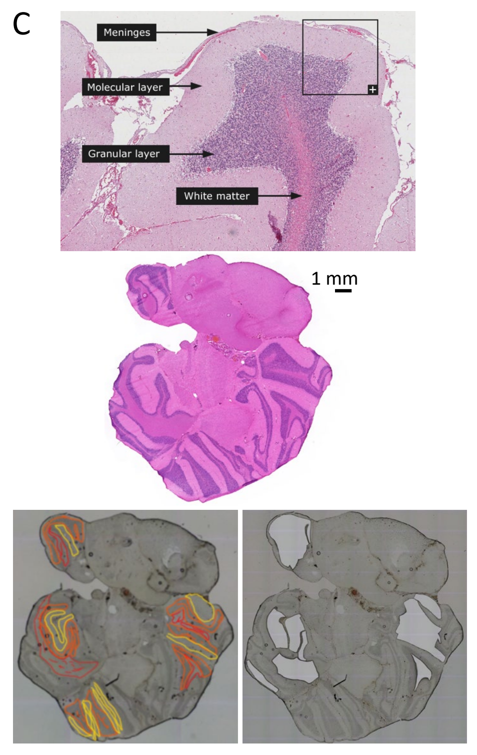

Supplemental Figure 2. CNS tissue processing post-necropsy for evaluation of bacterial burden, histopathology and spatial drug quantitation. **(A)** Macrodissection and gross appearance of CNS tissues sampled in this study. **(B)** Main features of the eight brain tissue slices collected for histology and laser-capture microdissection. **(C)** Example of laser-capture microdissection allowing isolation of anatomically distinct deep brain areas.

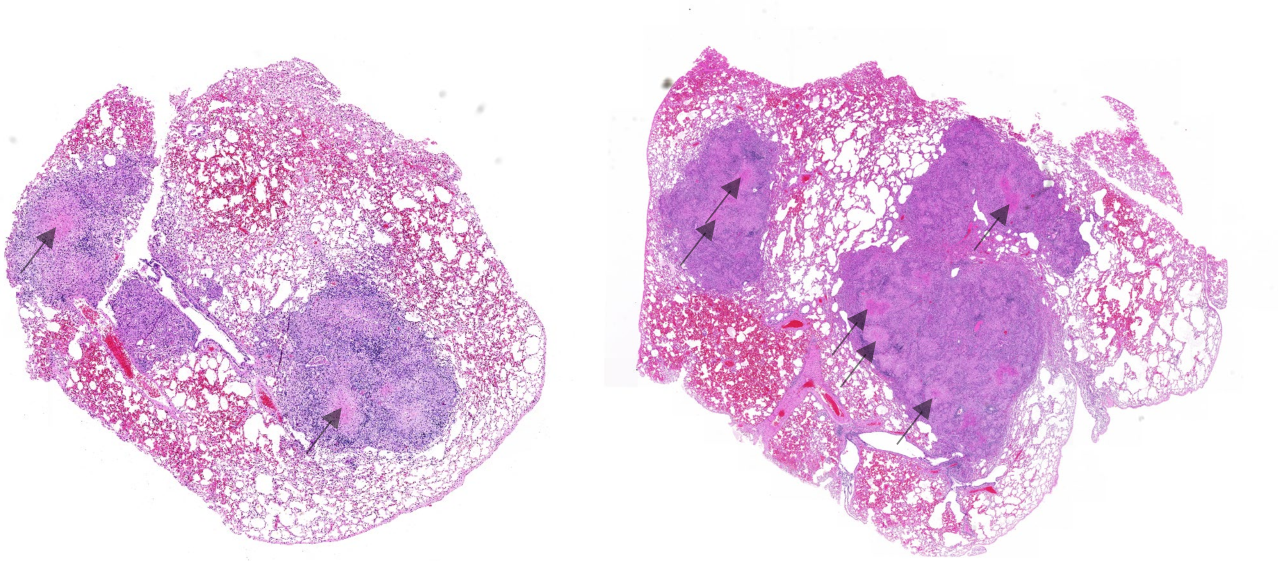

Supplemental Figure 3. Lung histopathology of representative rabbit 284 infected with 10^6 CFU from a frozen stock and reaching a neurological score of 4 after 13 weeks of CNS infection. H&E staining reveals substantial lung involvement and large areas of coalescing granulomas with multiple necrotizing foci (black arrows).

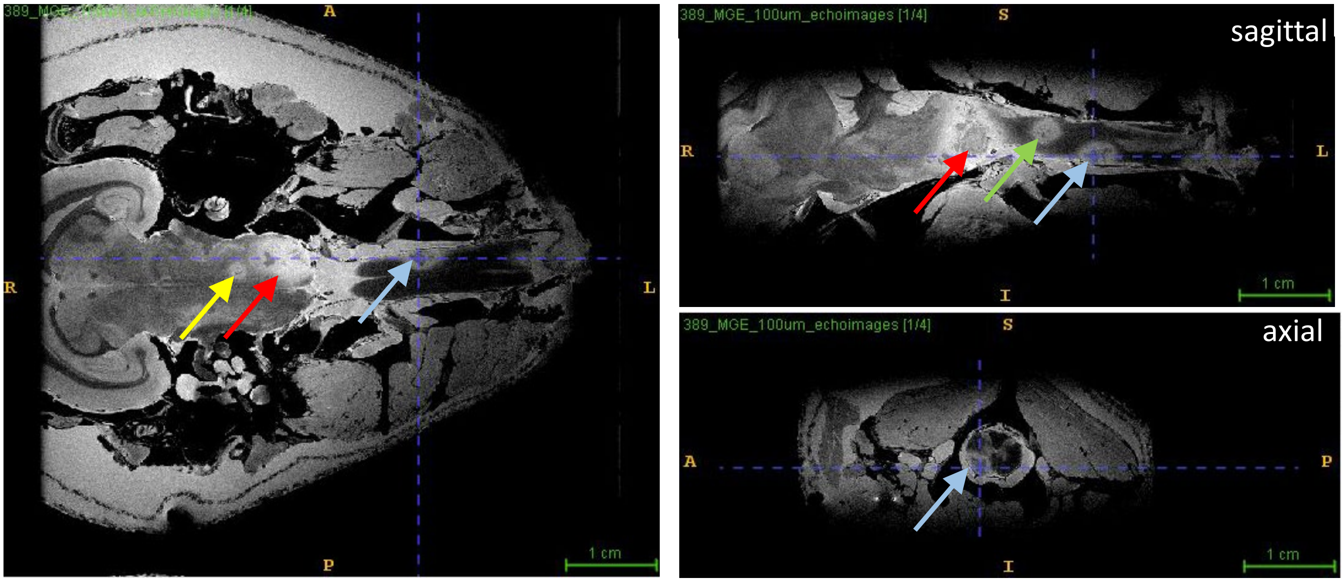

Supplemental Figure 4. Ex vivo, non contrast-enhanced, MRI of the cranial area, brain stem and cervical spine of rabbit 389. T2*-weighted (1) images taken along the coronal, sagittal and axial planes show mass lesions (arrows) in the brain stem and cervical spine area, compatible with tuberculomas (2, 3). The blue arrow points to the same lesion viewed in all 3 planes, showing central hypointensity and a hyperintense rim, compatible with a caseating tuberculoma with surrounding oedema (2, 4). Scale bar: 1 cm.

References

1. Chavhan GB, Babyn PS, Thomas B, Shroff MM, Haacke EM. 2009. Principles, techniques, and applications of T2*-based MR imaging and its special applications. Radiographics 29:1433-49.
2. Salvador GLO, Basso ACN, Barbieri PP, Leitao CA, Teixeira BCA, Neto AC. 2021. Central nervous system and spinal cord tuberculosis: Revisiting an important disease. Clin Imaging 69:158-168.
3. Katrak SM. 2021. Central nervous system tuberculosis. J Neurol Sci 421:117278.
4. Sadeghi N, Rorive S, Lefranc F. 2003. Intracranial tuberculoma: is diffusion-weighted imaging useful in the diagnosis? Eur Radiol 13:2049-50.
